# The relationships between serum fructosamine concentrations and lipid profiles in community-dwelling adults

**DOI:** 10.1038/s41598-017-07287-5

**Published:** 2017-07-31

**Authors:** You-Fan Peng, Ye-Sheng Wei

**Affiliations:** Department of Laboratory Medicine, Affiliated Hospital of Youjiang Medical University for Nationalities, Baise, Guangxi 533000 China

## Abstract

We examined the epidemiological associations between serum fructosamine and dyslipidemia indices in community-dwelling adults. Clinical characteristics and lipid profiles were analyzed in 1352 community-dwelling adults. The demographic characteristics and laboratory results were grouped according to the quartiles of serum fructosamine concentrations in all eligible individuals. From the bottom to the top quartile of serum fructosamine, there were graded increases in age, total cholesterol (TC), fasting blood glucose (FBG), total protein (TP), triglyceride (TG), total cholesterol/ high density lipoprotein-cholesterol (TC/HDL-C) and atherogenic index of plasma (AIP). Serum fructosamine was positive correlated with age, TC, FBG, TP, TG, TC/HDL-C and AIP in whole individuals. The positive correlations were then observed in both genders between serum fructosamine and TC, FBG, TP, TG. Two dominant factors were identified by principal component analysis. Logistic regression analysis showed that the two factors were associated with increased serum fructosamine with adjustment for gender, age, body mass index (BMI), FBG and TP. The similar results were observed in males, but not in females. Dyslipidemia tends to contribute to increased serum fructosamine concentrations in study population, suggesting that elevated serum fructosamine may herald an increased risk of cardiovascular disease among community-dwelling adults, especially in males.

## Introduction

The morbidity and mortality of cardiovascular disease are universal global events and are major challenges for public health, especially in developing countries^1^. Cardiovascular disease has been reported to be one of the most common and momentous public health crises worldwide^2^. The morbidity of cardiovascular disease accounts for approximately 66% in all disease diagnosis^3^. Serum fructosamine is a glycated protein to reflect average glycemia over the previous 2–3 weeks, and is considered to be a simple and inexpensive measure for the estimation of short to medium-term alteration in blood glucose concentrations^4^. Peng *et al*.^5^ reported a positive correlation between serum fructosamine and estimated glomerular filtration rate in non-diabetic individuals. Previous studies have demonstrated that higher serum fructosamine levels were associated with coronary artery atherosclerosis, dementia and renal failure^6–9^. Very recently, serum fructosamine has been found to be an independently predictor of vascular outcomes^10^. Accumulating data have shown that serum fructosamine is prospectively associated with risk of morbidity and mortality of cardiovascular disease in both diabetic and non-diabetic patients^11, 12^. Knowing that dyslipidemia is associated with significantly increased risk of cardiovascular disease. Up to our knowledge, the associations of serum fructosamine with lipid profiles in healthy population have been discussed poorly. In addition, serum fructosamine that serves as a reliable and indirect index to reflect lipid profiles may be valuable in healthy population. Therefore, we examined the epidemiological relationships between serum fructosamine and lipid profiles in community-dwelling adults.

## Materials and Methods

### Participants

A total of 1352 community-dwelling adults who underwent routine physical examinations in Affiliated Hospital of Youjiang Medical University for Nationalities between January 2015 and January 2016 were included in this cross-sectional study. All individuals were examined by general practitioners for physical examinations. Baseline demographics and medical history were obtained from medical-record department. Healthy individuals without hypertension, diabetes and stroke were eligible in our study. Additional exclusion criteria included known history of cardiovascular disease such as myocardial infarction, angina, coronary arterial disease, heart failure and atrial fibrillation. Moreover, individuals who contracted podagra, abnormal hepatic or renal function, infectious disease, autoimmune disease, malignant tumor, anemia, psychosis and pregnancy were also excluded. The research was executed on based of the Declaration of Helsinki, and all procedures were approved by the Ethics Committee of Affiliated Hospital of Youjiang Medical University for Nationalities, and informed consent was obtained in all participants.

### Biochemical assessment

Fasting venous blood samples were analyzed in clinical laboratory. Values of total cholesterol (TC), high density lipoprotein-cholesterol (HDL-C), low density lipoprotein-cholesterol (LDL-C), triglyceride (TG), fasting blood glucose (FBG), total protein (TP), and fructosamine were measured within 2 h after admission. Atherogenic index of plasma (AIP) was used to estimate a potential risk of atherosclerosis, the equation was log (TG/HDL-C)^13^. Weight and height were measured in all participants, and body mass index (BMI) was calculated by dividing weight in kilograms by height in meters squared (kg/m^2^). Plasma lipids were assayed by enzymatic methods, serum fructosamine was assayed by nitro blue tetrazolium chloride methods on automatic biochemical analyzer.

### Statistical analysis

Our data were analyzed by SPSS16.0 (SPSS Inc, Chicago, IL, USA) statistical software for statistical analysis. Continuous variables were shown as mean ± standard deviation, and categorical variables were presented as percentages. Chi-square test and one-way ANOVA test were used to examine differences in continuous and categorical variables. The correlations between the two continuous variables were analyzed using Spearman approach. Principal components analysis was used to determine factor loadings. The factor scores were considered as independent variable in logistic regression analysis. *P* < 0.05 was determined as significant.

## Results

Demographic characteristics and lipid profiles were analyzed in community-dwelling adults. The demographic characteristics and laboratory results were grouped according to the quartiles of serum fructosamine concentrations in all eligible individuals, as shown in Table 1. Mean values of TC, LDL-C, HDL-C, TG, TP, FBG, AIP and fructosamine were 4.8 ± 1.02 mmol/L, 2.5 ± 0.99 mmol/L, 1.4 ± 0.34 mmol/L, 1.8 ± 1.48 mmol/L, 72.0 ± 4.68 g/L, 5.1 ± 0.62 mmol/L, 0.06 ± 0.31% and 2.0 ± 0.35 mmol/L in all eligible individuals, respectively. From the bottom to the top quartile of serum fructosamine, there were graded increases in age, TC, FBG, TP, TG, TC/HDL-C and AIP.

**Table 1:** Demographic characteristics and lipid parameters results stratified by the quartiles of serum fructosamine (mmol/L) concentrations in community-dwelling adults.

Items	I ≤ 1.78 (N = 347)	II 1.78–1.92 (N = 346)	III1.92–2.31 (N = 327)	IV2.31 (N = 332)	*p-value*
Gender (male/female)	270/77	265/81	260/67	251/81	<0.001
Age (y)	54.6 ± 13.93	55.8 ± 14.32	56.9 ± 13.77	57.5 ± 14.16	0.038
Body mass index (Kg/m^2^)	23.9 ± 3.27	24.3 ± 2.89	24.1 ± 3.07	24.1 ± 2.83	0.352
TC (mmol/L)	4.6 ± 0.83	4.7 ± 1.03	4.8 ± 1.07	5.1 ± 1.07	<0.001
LDL-C (mmol/L)	2.5 ± 0.79	2.6 ± 0.91	2.5 ± 0.98	2.6 ± 1.25	0.565
HDL-C (mmol/L)	1.3 ± 0.31	1.4 ± 0.31	1.4 ± 0.35	1.4 ± 0.39	0.704
FBG (mmol/L)	5.1 ± 0.62	5.1 ± 0.57	5.2 ± 0.62	5.2 ± 0.64	0.001
TP (g/L)	71.3 ± 4.34	72.0 ± 4.72	71.4 ± 4.86	73.1 ± 4.59	<0.001
TG (mmol/L)	1.5 ± 0.73	1.6 ± 0.88	1.8 ± 1.28	2.3 ± 2.38	<0.001
TC/HDL (ratio)	3.6 ± 0.94	3.7 ± 1.09	3.7 ± 1.23	4.0 ± 1.40	<0.001
AIP (ratio)	0.01 ± 0.25	0.04 ± 0.28	0.05 ± 0.32	0.13 ± 0.38	<0.001

TC** = **Total cholesterol, LDL-C = Low density lipoprotein cholesterol, HDL-C = High density lipoprotein cholesterol, FBG = fasting blood glucose, TP = Total protein, TG = Triglycerides, TC/HDL = Total cholesterol/ high-density lipoprotein-cholesterol, AIP = Atherogenic index of plasma.

The results of analysis on the correlations between serum fructosamine and lipid profiles showed that serum fructosamine was positive correlated with age (r = 0.090, P = 0.001), TC (r = 0.172, P < 0.001), FBG (r = 0.115, P < 0.001), TP (r = 0.112, P < 0.001), TG (r = 0.143, P < 0.001), TC/HDL-C (r = 0.106, P < 0.001) and AIP (r = 0.110, P < 0.001) in whole individuals. The positive correlations were then observed in both genders between serum fructosamine and TC (r = 0.155, P < 0.001 in males; r = 0.229, P < 0.001 in females), FBG (r = 0.118, P < 0.001 in males; r = 0.128, P = 0.025 in females), TP (r = 0.087, P = 0.005 in males; r = 0.195, P < 0.001 in females), TG (r = 0.150, P < 0.001 in males; r = 0.159, P = 0.005 in females). In addition, serum fructosamine was found to be positive correlated with AIP (r = 0.131, P < 0.001) in males.

To identify the main contributors of the lipid profiles for serum fructosamine levels in the study population, the lipid variables (TC, LDL-C, HDL-C, TG, TC/HDL-C, AIP) were investigated by principal component analysis with varimax (orthogonal) rotation. The lipid variables were combined into factors, two dominant factors were identified by principal component analysis, subsequently named the “AIP” pattern (factor 1) and the “LDL-C” pattern (factor 2), as shown in Table 2, the total variance was explained by the two factors was 82.4%. Individual factor scores were obtained for each pattern by summing the original lipid variables multiplied by their factor loadings. To investigate the relationships between the two patterns (factor 1 and factor 2) and serum fructosamine, the two factor scores were considered as independent variable in the logistic regression model predicting serum fructosamine levels. Logistic regression analysis showed that the two factors were associated with increased serum fructosamine with adjustment for gender, age, BMI, FBG and TP, the resulting odds ratio (OR) and 95% confidence interval (95% CI) of fourth vs first quartile of serum fructosamine were 1.676 (95% CI: 1.405–1.999, P < 0.001) for factor 1, 1.266 (95% CI: 1.070–1.499, P = 0.006) for factor 2 (Table 3).

**Table 2:** Principal component loadings demonstrating importance of each variable in accounting for the variability in principal component analysis.

Items	Component 1	Component 2
Total cholesterol	0.423	0.844
Low density lipoprotein cholesterol	0.083	0.954
High density lipoprotein cholesterol	−0.702	0.338
Triglycerides	0.791	−0.280
Total cholesterol/high density lipoprotein-cholesterol	0.916	0.278
Atherogenic index of plasma	0.919	−0.253

**Table 3:** Associations between dominant factors and serum fructosamine in whole population in logistic regression analysis (1^st^ versus 4^th^ quartile).

ltems	B	SE	Wald	P-value	OR	95%CI
Age	0.024	0.007	13.130	<0.001	1.024	1.011–1.037
Gender	−0.493	0.210	5.498	0.019	0.611	0.404–0.922
Body mass index	0.038	0.027	1.933	0.164	1.039	0.985–1.096
Fasting blood-glucose	0.108	0.141	1.418	0.234	1.183	0.897–1.561
Total protein	0.095	0.020	23.449	<0.001	1.024	1.011–1.037
Factor 1	0.516	0.090	33.056	<0.001	1.676	1.405–1.999
Factor 2	0.236	0.086	7.515	0.006	1.266	1.070–1.499

Since physiological characteristics are different between females and males, so that we decided to separate the population into genders for further statistical analysis. In male individuals (fourth vs first quartile of serum fructosamine as dependent variable), the two factors were found to be independently associated with serum fructosamine in logistic regression analysis (OR = 1.804, 95% CI: 1.464–2.223, P < 0.001 for factor 1; OR = 1.230, 95% CI: 1.012–1.493, P = 0.037 for factor 2). Nevertheless, the similar results were not observed in females, as shown in Tables 4 and 5.

**Table 4:** Associations between dominant factors and serum fructosamine in males in logistic regression analysis (1^st^ versus 4^th^ quartile).

ltems	B	SE	Wald	P-value	OR	95%CI
Age	0.018	0.007	6.280	0.012	1.018	1.004–1.033
Body mass index	0.021	0.032	0.444	0.505	1.021	0.960–1.087
Fasting blood-glucose	0.248	0.155	2.544	0.110	1.282	0.945–1.738
Total protein	0.086	0.023	14.653	<0.001	1.090	1.043–1.139
Factor 1	0.590	0.107	30.653	<0.001	1.804	1.464–2.223
Factor 2	0.207	0.099	4.346	0.037	1.230	1.012–1.493

**Table 5:** Associations between dominant factors and serum fructosamine in females in logistic regression analysis (1^st^ versus 4^th^ quartile).

ltems	B	SE	Wald	P-value	OR	95%CI
Age	0.070	0.019	13.708	<0.001	1.073	1.034–1.114
Body mass index	0.102	0.057	3.205	0.073	1.107	0.990–1.238
Fasting blood-glucose	−0.201	0.371	0.293	0.588	0.818	0.395–1.694
Total protein	0.120	0.041	8.564	0.003	1.128	1.040–1.212
Factor 1	0.083	0.203	0.167	0.683	1.087	0.730–1.618
Factor 2	0.169	0.188	0.812	0.367	1.114	0.820–1.711

## Discussion

This study demonstrated the strong relationships between serum fructosamine and lipid profiles in study population, the results showed that dyslipidemia might tend to increase serum fructosamine among community-dwelling population, especially in man. Dyslipidemia characterized by higher TC, TG, LDL-C and lower HDL-C is crucial risk factor of atherosclerosis, stroke and cardiovascular disease^14^. Some earlier investigations have shown that dyslipidemia is associated with increased risk of cardiovascular events in patients with diabetes^15^. Our study observed the associations between serum fructosamine and lipid profiles in study population, suggesting serum fructosamine may provide an additional information for dyslipidemia in community-dwelling adults. There is growing evidence that modified LDL is trapped in the extracellular matrix of endothelial tissue, which is a major factor in the processes of atherosclerosis^16^. LDL oxidation has been regarded as an importantly initiator factor of atherosclerosis^17^. In addition, the interactive relationship between oxidative stress and lipid has been reported in previous studies^18^, and lipid peroxidation products can induce oxidative stress^19^. Turkdogan KA *et al*.^18^ reported that higher levels of lipid, even at an early age, might promote oxidative stress in healthy subjects. These studies mentioned above indicated a potential relationship between oxidative stress and dyslipidemia. Unfortunately, oxidative stress in fact influences protein glycation^20^, and there is point that oxidative stress is a key mechanism for albumin glycation in individuals without diabetes^21^. Thus, oxidative stress induced by dyslipidemia is a possible explanation regarding the mechanism between serum fructosamine and lipid profiles. On the other hand, a positive relation between serum fructosamine and TP levels was suggested by Shafi T *et al*.^22^. In agreement with the results, the present study observed the relationship of serum fructosamine with TP in whole participants, including males and females. Recently, albumin is served as a carrier of insulin-sensitising signal transduction, and is associated with physiological insulin sensitivity^23^. Historically, previous studies showed that serum fructosamine is correlated with increased insulin resistance in subjects with and without diabetes^24^. Obviously, these results may contribute to explain the correlation of serum fructosamine with TP. The formation of advanced glycation end products occurs by multiple processes related with reactive oxygen species^25^, thus, a complementary explanation to the positive correlation between serum fructosamine and age is given by the possible contribution of age-associated chronic inflammation in accelerating albumin catabolism^23^. However, the further pathophysiologic mechanism for these associations in the study population is needed with more investigation.

Very recently, Zaccardi F *et al*.^26^ have focused on the relationship between serum fructosamine and risk of cardiovascular in men living in communities, they found that the all-cause mortality of cardiovascular disease were not independently associated with serum fructosamine. In contrast, a cohort study provides an evidence that serum fructosamine was linked to an increase in the risk of vascular outcomes and mortality in community residents^27^. Similarly, higher serum fructosamine levels have been demonstrated to be associated with the morbidity of microvascular complications in non-diabetic individuals^28^. In earlier research, Misciagna G^11^ reported that non-enzymatic glycation of protein was used to predict cardiovascular events in non-diabetic subjects. In our study, we found that higher serum fructosamine levels were associated with lipid metabolism disorders in men, which indicates serum fructosamine may predict an increased risk of cardiovascular disease in men. This contradiction may be explained by following points: First, the differences derived from the heterogeneity of populations may be an important confounder in resulting in the problem. Moreover, diet style in different regions and populations may influence metabolic profiles, which may be related to the presence of cardiovascular disease in general population^29^. Finally, the different duration of follow-up possibly leads to the differences between these outcomes in various studies.

In the current study, we should acknowledge, however, several limitations and strengths. First, the lack of evidence linking serum fructosamine to long-term outcomes of cardiovascular disease was a major limit. Second, a relationship between glycosylated hemoglobin and lipid profiles was not assessed in the present study. Third, only single measures of laboratory parameters were carried out in our laboratory. Finally, our study only included healthy subjects, so that the conclusions may be limited by the extrapolation of results in hospitalized patients. In our study, dyslipidemia tends to contribute to increased serum fructosamine concentrations in study population, suggesting that elevated serum fructosamine may herald an increased risk of cardiovascular disease among community-dwelling adults, especially in males.
